# Thoracoscopic repair of penetrating lung trauma with pulmonary tractotomy: a case report

**DOI:** 10.1186/s44215-025-00209-2

**Published:** 2025-05-26

**Authors:** Hiroyasu Koga, Katsuya Watanabe, Aya Saito

**Affiliations:** 1 Department of Thoracic Surgery, Yokohama Medical Center, 3-60-2 Harajuku, Totsuka-Ku, Yokohama, Kanagawa 245-8575 Japan; 2 Department of Surgery, Yokohama City University, 3-9 Fukuura, Kanazawa-Ku, Yokohama, Kanagawa 236-0004 Japan

**Keywords:** Pulmonary tractotomy, Thoracoscopic surgery, Penetrating lung injury, Chest trauma, Pneumothorax, Hemothorax, Contaminated wound

## Abstract

**Background:**

Penetrating lung injuries are rare but potentially life-threatening. Pulmonary tractotomy (PT) allows for repair while preserving lung parenchyma. This case demonstrates the effectiveness of thoracoscopic PT.

**Case report:**

An 81-year-old woman sustained a right chest stab wound with a gardening stake. Imaging revealed a right pneumothorax without cardiac or major vascular injury. A chest tube was inserted, draining blood-stained fluid and revealing a persistent air leak. Progressive anemia necessitated transfusion, and surgery was planned due to suspected contamination. Thoracoscopy identified a 1.5 cm fistula in the S3 segment of the right upper lobe extending toward S2. A stapler was inserted to perform tractotomy, revealing a bronchial injury, which was sutured. The air leak resolved completely. The postoperative course was uneventful. Empirical antibiotics were administered for 5 days. The chest tube was removed on day 5, and the patient was discharged on day 13.

**Conclusion:**

Thoracoscopic PT is considered a safe and effective option for selected penetrating lung injuries, preserving lung function and reducing surgical trauma. Careful preoperative imaging and intraoperative bronchial evaluation are essential for safe and effective thoracoscopic PT.

## Background

Thoracic trauma is a critical condition frequently encountered in emergency respiratory surgery. Compared to injuries in other regions, thoracic trauma has a higher proportion of severe cases, and sharp thoracic injuries often require urgent surgical intervention. In this case, surgery was deemed necessary to address a penetrating lung injury caused by a stake, considering the risks of bleeding, air leakage, and infection. We performed pulmonary tractotomy (PT), a minimally invasive procedure, under thoracoscopic guidance, and the patient had a favorable postoperative course.

## Case report

An 81-year-old woman with a medical history of hypertension under treatment presented with a stab wound to the right chest. She was stabbed with a gardening stake during an argument with her son and was found collapsed at the entrance of her home by a neighbor, who called emergency services. Upon arrival at the hospital, her Glasgow Coma Scale (GCS) score was E1 V2M4, body temperature was 34.9 °C, blood pressure was 60/40 mmHg, heart rate was 90/min, respiratory rate was 24/min, and SpO_2_ was 90% on 10L oxygen via face mask. Physical examination revealed multiple lacerations and stab wounds on her face, upper limbs, and right chest, though the stake was no longer present, and there was no active external bleeding. Breath sounds were diminished on the right side, but no jugular vein distension or subcutaneous emphysema was observed. Laboratory findings showed no significant abnormalities in blood counts, biochemistry, or coagulation tests.

**Fig. 1:**
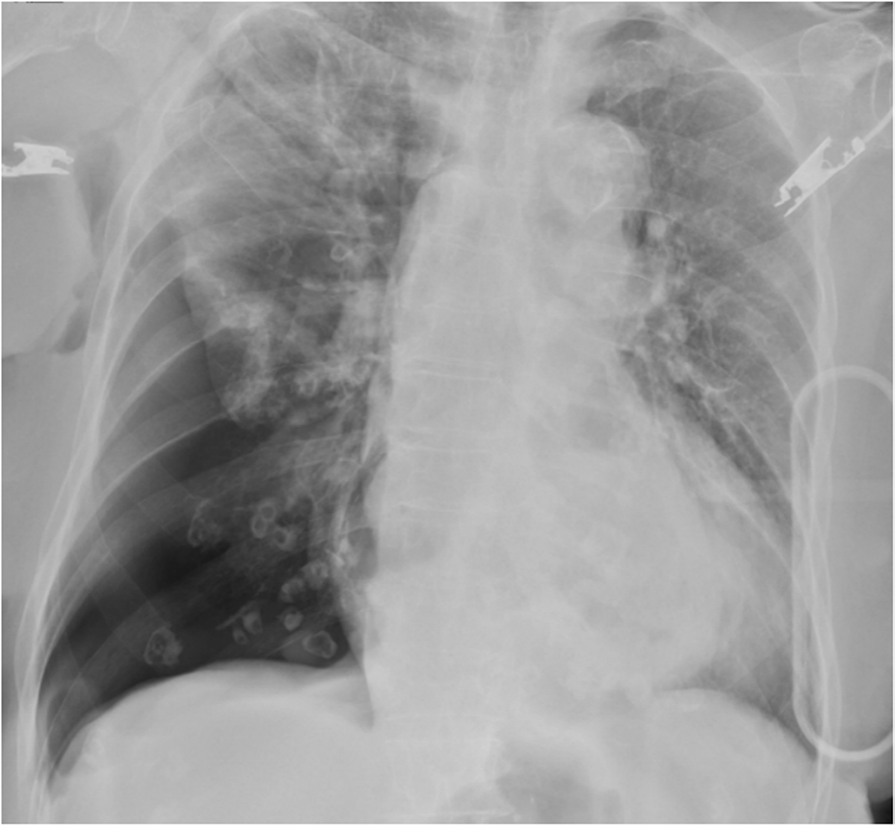
Chest X-ray reveals lung collapse in the right lower lung field, with a possible adhesion of the upper lobe to the chest wall

**Fig. 2:**
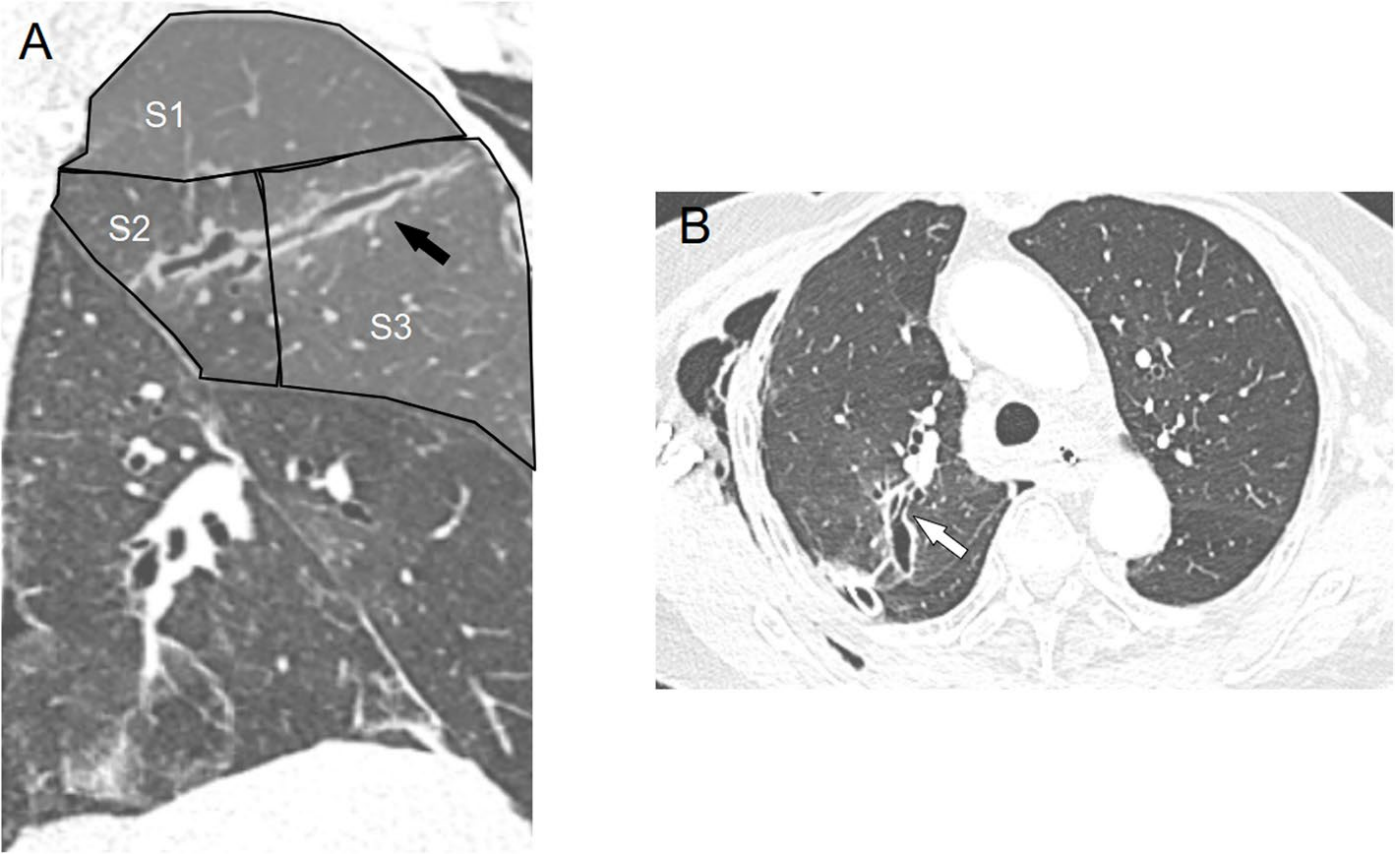
Chest CT shows no evidence of cardiac injury, major vessel injury, or intrapulmonary hematoma. **A** Axial view reveals a cavitary lesion in the right upper lobe communicating with the B2 bronchus (black arrow). B Sagittal view shows a cord-like shadow extending from S3 to S2 (white arrow)

**Fig. 3:**
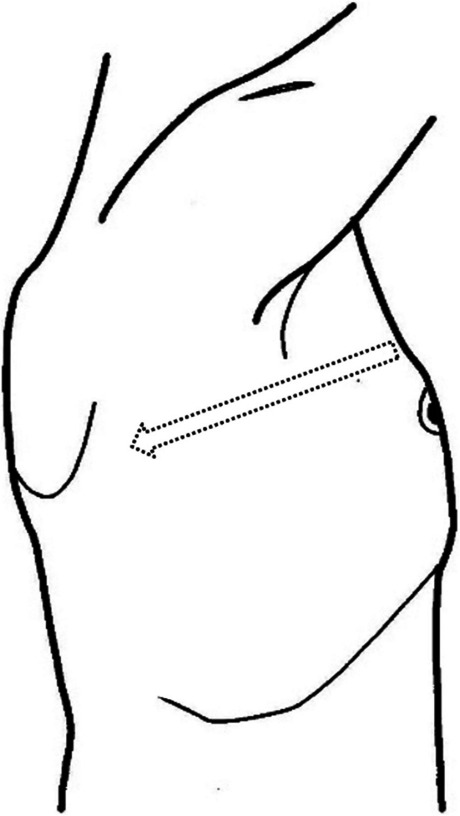
A schematic diagram of the torso is shown. Based on the external wound and chest CT findings, it suggests that the stake had penetrated from the anterior chest wall toward the posterior direction (dotted arrow)

The chest X-ray (Fig. 1) showed a collapsed right lung. The contrast-enhanced computed tomography (CT) included a sagittal view (Fig. 2A) showing a cord-like cavitary shadow extending from S3 to S2 in the upper lobe (black arrow), and an axial view (Fig. 2B) indicating a bronchial injury at B2 (white arrow), with no evidence of major vascular injury. Figure 3 shows a schematic diagram of the torso, suggesting that the stake had penetrated into the thoracic cavity, as indicated by the dotted arrow. The patient was diagnosed with traumatic hemothorax and pneumothorax caused by a penetrating lung injury. A 24-Fr chest tube was inserted into the right pleural cavity, which drained blood-stained fluid and revealed a persistent air leak. Chest tube insertion alleviated the hemodynamic compromise due to pneumothorax and led to initial improvement of the shock state. Approximately 800 mL of bloody pleural fluid was drained within the first 12 h. The patient’s hemoglobin level decreased from an initial value of 12 g/dL to the 6 g/dL range, prompting transfusion of 6 units of packed red blood cells (RBC) and 6 units of fresh frozen plasma (FFP). Despite transfusion, the hemoglobin level only increased to a maximum of 8.0 g/dL, and persistent bloody drainage from the chest tube was observed. Considering the nature of the injury involving a gardening stake, Sulbactam/Ampicillin (Sultamicillin) was administered as empirical antibiotic therapy to cover common skin flora, gram-negative organisms, and anaerobes. Tetanus toxoid was also administered. Although pleural fluid was visually inspected intraoperatively, no purulent fluid was found and bacterial culture was not performed. Based on these findings, surgical intervention was planned to address bleeding, air leakage, and potential infection. Based on the absence of central pulmonary vascular injury or intrapulmonary hematoma on imaging, the clearly localized injury site on CT, and stable vital signs following initial treatment, a minimally invasive approach using video-assisted thoracoscopic pulmonary tractotomy (VATS-PT) was selected to preserve lung parenchyma. Preparations were also made to allow immediate conversion to open thoracotomy in case of unexpected intraoperative bleeding.

**Fig. 4:**
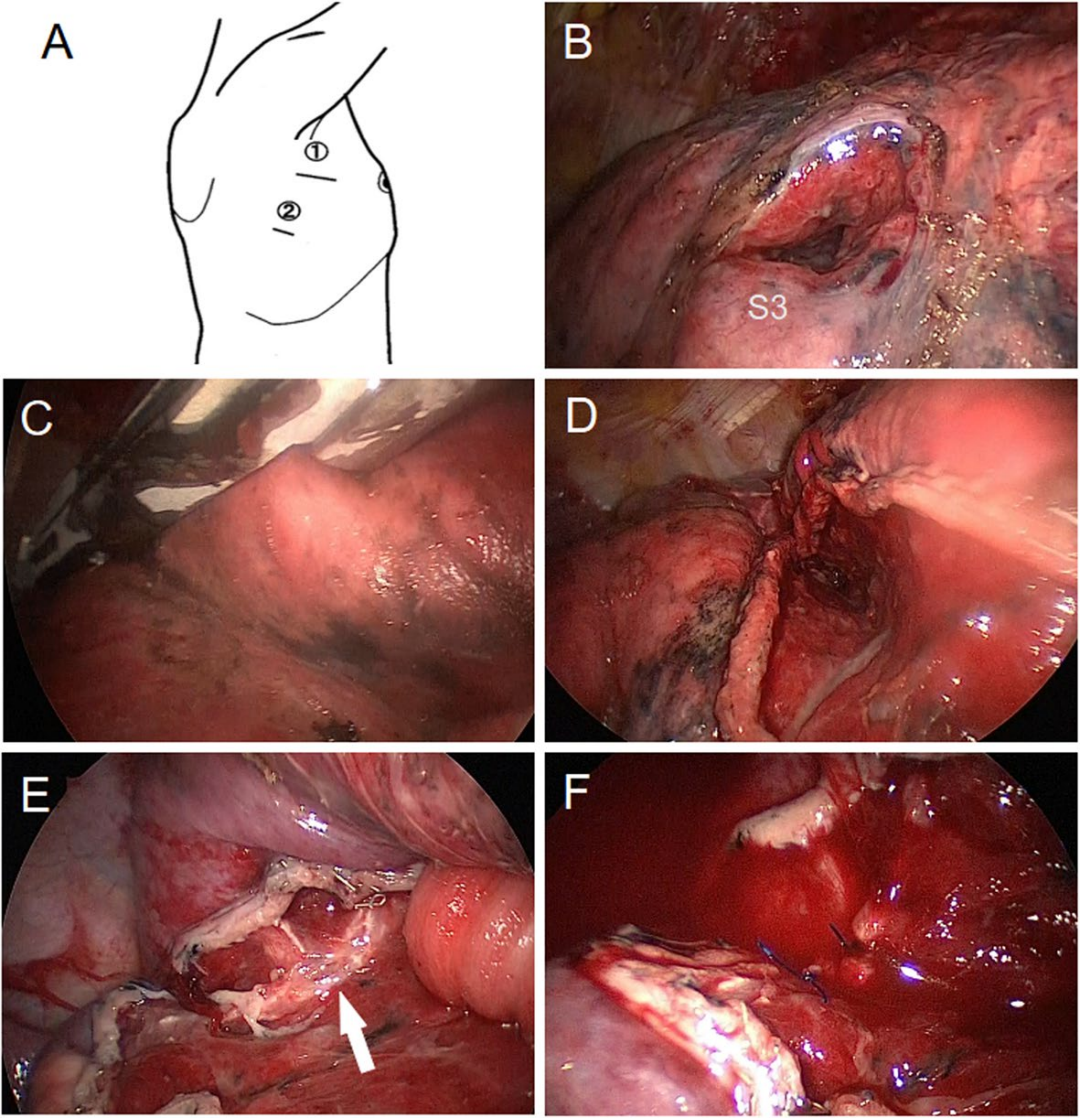
Thoracoscopic surgery was performed using two ports in the 4 th and 6 th intercostal spaces (**A**). A fistula approximately 1.5 cm in size was identified in the right lung's S3 segment (**B**). One blade of a stapler was inserted toward the S2 segment (**C**), and the area was transected and opened (**D**). Damage to a peripheral branch of B2 (arrow) was found, accompanied by an air leak (**E**). The air leak was resolved after suturing and closing the affected area (**F**)

A 5 cm thoracotomy was performed in the right 4 th intercostal space, and a camera port was inserted at the mid-axillary line in the right 6 th intercostal space (Fig. 4A). Thoracoscopic examination revealed a 1.5 cm fistula in the S3 segment of the right upper lobe, presumed to be the entry point (Fig. 4B). No active bleeding was observed in the thoracic cavity, and a clamp was easily passed through the fistula without resistance. The fistula extended toward the S2 interlobar plane, but no exit wound was identified. A linear stapler was carefully inserted along the fistula (Fig. 4C) to divide and open the pulmonary parenchyma (Fig. 4D). After thorough irrigation of the thoracic cavity, a sealing test revealed air leakage from a bronchial fistula involving the distal branch of B2 (Fig. 4E). The air leak was successfully controlled by suturing the fistula (Fig. 4F). A single chest tube was placed at the apex of the lung, and the external wound was irrigated and closed. The total operative time was 123 min, blood loss was 50 mL, and ephedrine was transiently administered intraoperatively. No vasopressors were required postoperatively, and the patient was weaned from mechanical ventilation in the operating room.

**Fig. 5:**
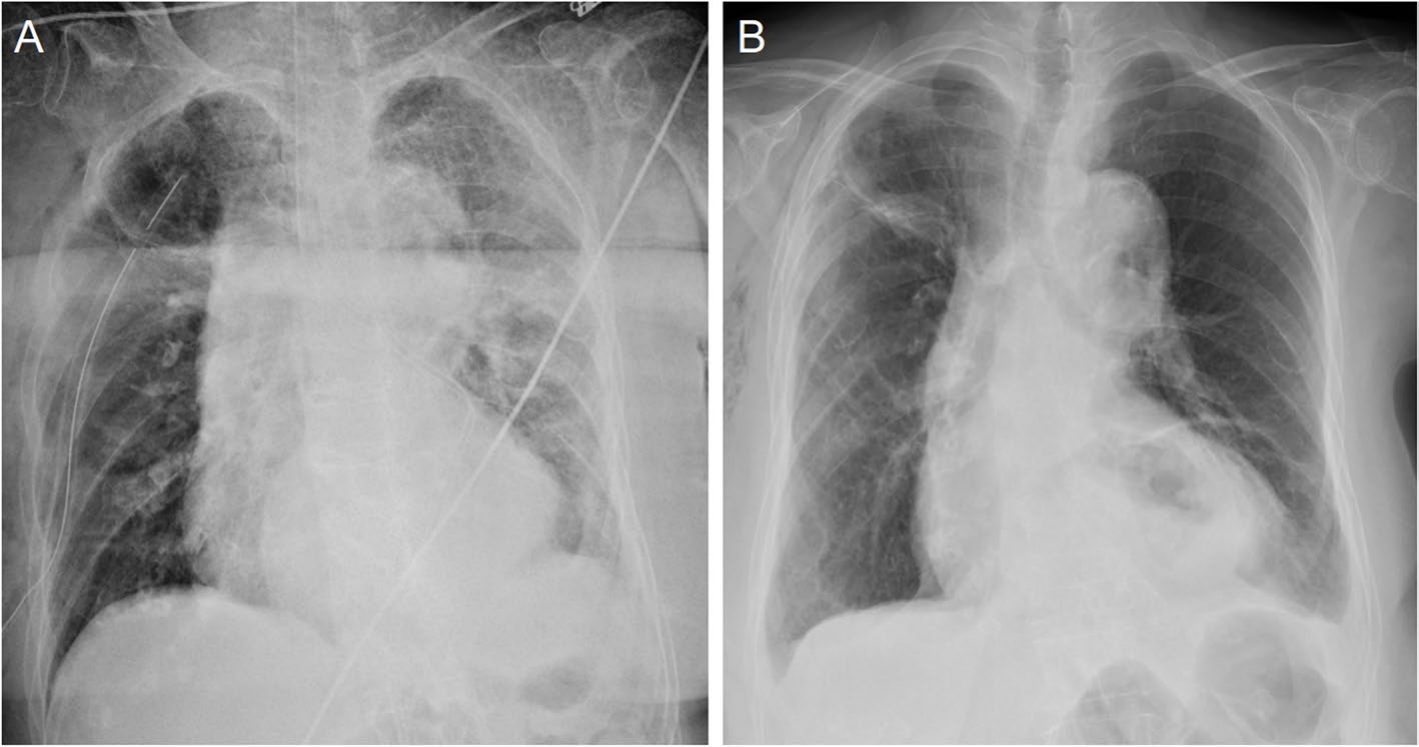
The chest X-ray on postoperative day 1 shows good expansion of the right lung. On postoperative day 9, the X-ray reveals a shadow at the resection site in the right upper lung, but there is no recurrence of pneumothorax

The postoperative course was uneventful. There were no signs of bleeding or air leakage, and inflammatory markers showed an improving trend. Antibiotic therapy was completed on postoperative day 4, with a total duration of 5 days, and the chest tube was removed on postoperative day 5. The patient was transferred to a long-term care facility on postoperative day 13 (Fig. 5).

## Discussion

In Japan, the incidence of penetrating trauma remains relatively low, accounting for approximately 3.7% of all injuries [1]. Particularly, due to strict regulations on firearm possession, gunshot wounds are extremely rare, comprising only 0.04% of all trauma cases [2]. In contrast, stab wounds to the chest are occasionally encountered, among which impalement injuries are considered relatively uncommon, representing about 0.15% of all reported trauma cases [2].

Impalement injuries differ from conventional stab wounds caused by bladed weapons in several important aspects. First, the trajectory of the penetrating object is often irregular and complex [3]. Second, mechanical pressure exerted during penetration tends to cause extensive tissue damage across a wide area [4]. Third, the presence of retained foreign bodies or rusted metallic fragments significantly increases the risk of infection [5–7]. Given these characteristics, surgical treatment for impalement injuries—particularly those involving penetrating lung trauma—must be carefully selected based on the location and extent of injury, as well as the patient's overall condition. Successful management hinges on effectively addressing key challenges, including control of bleeding and air leaks, prevention of infection, and preservation of lung function. In the present case, preservation of pulmonary function was prioritized, and pulmonary tractotomy (PT) was performed thoracoscopically.

Pulmonary tractotomy (PT) was first described by Wall et al. in 1994 as a technique for repairing lung injuries, and its efficacy has since been validated in numerous studies [8]. This procedure involves opening the injured area to directly control bleeding and air leaks, thereby minimizing lung parenchymal resection and preserving postoperative respiratory function. By avoiding closure of the wound and instead leaving it open, PT also reduces the risk of postoperative complications such as empyema and lung abscess, representing a significant advantage over anatomical lung resection. In recent years, the use of automatic staplers for controlled tract opening has been reported [9–11], with some reports supporting the enhanced safety of using Penrose drains as stapler guides during tractotomy [12].

With regard to thoracoscopic approaches for penetrating lung trauma, there are several reports of successful thoracoscopic removal of intrapulmonary foreign bodies [13, 14], as well as reports of PT combined with free subcutaneous fat pad coverage under thoracoscopic guidance [15]. A search of PubMed using the terms “pulmonary tractotomy” and “thoracoscopy” from 1994 (the year of PT’s first description by Wall) to March 2025 yielded no reports of fully thoracoscopic PT. Thus, to the best of our knowledge, this case may represent the first documented instance of thoracoscopic pulmonary tractotomy performed entirely under thoracoscopic visualization.

In our case, we selected a minimally invasive approach via video-assisted thoracoscopic surgery for PT (VATS-PT), based on the absence of central pulmonary vascular injury or intrapulmonary hematoma on imaging, the clear localization of the injury on CT, and the patient’s stable vital signs. A stapler was successfully inserted along a pre-planned route to complete the tractotomy. Manlulu et al. reported performing VATS in 19 cases of hemodynamically stable thoracic trauma, with no conversions to thoracotomy [16]. Conversely, other reports indicate that up to 24% of VATS procedures required conversion to open thoracotomy [17]. These findings suggest that indications for VATS in trauma patients must be considered with caution, particularly in relation to hemodynamic stability. In our case, although the patient was stable preoperatively, full preparations were made for immediate thoracotomy in case of unexpected intraoperative bleeding.

The successful implementation of PT requires detailed preoperative evaluation of the injury and vascular anatomy via CT, enabling careful planning of the incision to avoid major pulmonary vessels and minimize the risk of ischemia or necrosis. In this case, the injury and surrounding vasculature were accurately assessed on CT, allowing for a safe and effective tractotomy. Compared to thoracotomy, VATS offers several advantages, including improved postoperative pain control, better lung re-expansion, earlier drain removal, and faster return to daily activities [18]. The combination of VATS and PT allowed for a minimally invasive, lung-sparing approach even in an elderly patient, contributing to early postoperative recovery.

In conclusion, pulmonary tractotomy (PT) represents a viable lung-preserving surgical alternative to lung resection for relatively mild penetrating lung injuries without major involvement of hilar structures. This case is particularly rare in that thoracoscopic PT along with bronchial fistula closure, was successfully performed, preserving pulmonary function and achieving early recovery without complications in an elderly patient. Safe implementation of thoracoscopic PT requires precise preoperative imaging to assess the extent of injury and vascular anatomy, careful intraoperative technique, and preparedness for emergent conversion in the event of unexpected bleeding or complications. With the accumulation of future case reports and clinical studies, the indications and clinical utility of thoracoscopic PT are expected to become further clarified.

## Data Availability

In this study, no data were generated or analyzed, and therefore, data sharing is not applicable.

## Abbreviations

PT: Pulmonary tractotomy
AP: Antibiotic prophylaxis
CT: Computed tomography
GCS: Glasgow Coma Scale
SpO_2_: Peripheral capillary oxygen saturation
B#: Segmental Bronchus (e.g., B2: Segmental Bronchus 2)
S#: Pulmonary Segment (e.g., S2: Posterior Segment, S3: Anterior Segment)
